# Editorial: Intracellular bacterial pathogens: Infection, immunity and interventions

**DOI:** 10.3389/fvets.2023.1163008

**Published:** 2023-03-13

**Authors:** Subhash Verma, Aneesh Thakur

**Affiliations:** ^1^Department of Veterinary Microbiology, Dr. G. C. Negi College of Veterinary and Animal Sciences, CSK Himachal Pradesh Agricultural University, Palampur, Himachal Pradesh, India; ^2^Vaccine and Infectious Disease Organization, University of Saskatchewan, Saskatoon, SK, Canada

**Keywords:** *Brucella*, *Mycobacterium avium* subspecies *paratuberculosis* (MAP), correlates of protection, small regulatory RNA, *Ehrlichia*

Many intracellular bacterial pathogens belonging to a variety of genera including *Brucella, Mycobacterium, Ehrlichia*, and *Chlamydia*, not only affect animals but also have a zoonotic potential and, thus, pose a substantial global one health threat (1). It is estimated that ~1 billion people are at risk of infection with intracellular bacterial pathogens and these infections lead to substantial economic losses in production animals (2). There are several aspects of these pathogens that still remain unclear such as host–pathogen interaction, co-infections, intracellular survival, evasion of host immune responses, and variable disease outcomes (3).

The current Research Topic “*Intracellular bacterial pathogens: Infection, immunity and interventions*” dwells on some important aspects of a few of these pathogens. In this special collection, four out of six articles were accepted.

The only article in the form of a review by King et al. presented a comprehensive summary of the role of Hfq chaperone and small regulatory RNAs (sRNAs) in the *Brucella* physiology and virulence, particularly the organism's survival within the macrophages by referring to various studies (4–7). More specifically, they have drawn the attention of the readers to the absence of a uniform, well-accepted nomenclature for describing sRNAs and the difficulty it poses to compare their regulatory functions across different species of *Brucella*. The authors have proposed to designate new sRNAs, as they are being identified with the acronym “Bsr” for *Brucella* sRNA, regardless of the species in which the sRNA is identified. The need to elucidate the sRNAs-mediated pathways in the brucellae was also emphasized.

The next two articles address important gaps in our current knowledge with respect to the disease pathogenesis and vaccine development against *Mycobacterium avium* subspecies *paratuberculosi*s (MAP). The MAP is responsible for causing chronic debilitating enteritis known as Johne's disease in ruminants that accrue substantial losses to the livestock industry globally (8, 9).

The first article by Purdie et al. reported important gene transcripts as correlates of vaccine protection following vaccination of sheep with the commercial Johne's disease vaccine Gudair^®^ using a transcriptomic approach; whereas, the second article by Blake et al. described the development of 3D bovine intestinal organoid to understand host–pathogen interaction and pathogenesis of MAP.

Having a thorough understanding of the correlates of protection helps in designing better vaccines (10). For a majority of the currently approved vaccines, antibodies in the serum or mucosa correlate with disease protection and are quantified by using ELISA, neutralization, and phagocytic assays. The first article presented a transcriptomics approach to identify vaccine-induced correlates for protection. Since the genes were differentially expressed between sheep with protective vs. unprotected responses, these genetic correlates have the potential as tools for the identification and culling of poor vaccine responders to curtail losses.

The second article details the development and characterization of physiologically relevant *in vitro* 3D bovine intestinal organoid models for their application to investigate MAP pathogenesis and to understand host–pathogen interaction in the small intestine without the need for a target host to carry out experimental studies. They have achieved this by challenging these enteroid-derived models of the bovine intestine with a laboratory reference strain and a field MAP isolate and by using a combination of techniques such as confocal microscopy and qRT-PCR.

The last article by Urbani et al. described the clinical presentation, pathogenicity, and therapeutic response in tick-borne *Ehrlichia canis* and *Hepatozoon canis* in concomitant infection of canine parvovirus in dogs. The study also emphasized the need to screen and manage infections such as ehrlichiosis etc. when dogs come from vector-borne pathogens endemic regions.

In summary, the results of the above-mentioned studies and reviews represent new findings on the host–pathogen interaction, disease pathogenesis, and vaccine-induced correlates of protection against some intracellular pathogens of veterinary importance that have zoonotic potential. The articles published in this Research Topic highlight the application of cutting-edge methods such as transcriptomics and organoids to study disease pathogenesis and evaluation of vaccine efficacy, which introduces the reader to new avenues to explore host–pathogen interaction and the development of novel therapeutics and prophylactics against intracellular pathogens.

## Author contributions

SV wrote the introduction and the central part. AT wrote the conclusion and contributed to the central part with comments on the cited articles and references. Both authors contributed to the manuscript and approved the submitted version of the manuscript.
